# Primary explants of the postnatal thymus allow the expansion of clonogenic thymic epithelial cells that constitute thymospheres

**DOI:** 10.1186/s13287-023-03529-8

**Published:** 2023-10-31

**Authors:** Juan M. Ocampo-Godinez, Jose L. Gonzalez-Quiroz, Hector Cote-Palafox, Elizabeth George, Jael A. Vergara-Lope Nuñez, Guillermo Villagomez-Olea, Febe C. Vazquez-Vazquez, Edgar O. Lopez-Villegas, Gloria Leon-Avila, Maria L. Dominguez-Lopez, Marco A. Alvarez-Perez

**Affiliations:** 1 Laboratorio de Bioingeniería de Tejidos, División de Estudios de Posgrado e Investigación, Universidad Nacional Autónoma de Mexico, Mexico City, Mexico; 2 Laboratorio de Genética, Departamento de Zoología, Escuela Nacional de Ciencias Biológicas, Instituto Politécnico Nacional, Mexico City, Mexico; 3 Carrera de Médico Cirujano, Facultad de Estudios Superiores Iztacala, Universidad Nacional Autónoma de Mexico, Mexico City, Estado de Mexico Mexico; 4 Laboratorio de Inmunoquímica I, Departamento de Inmunología, Escuela Nacional de Ciencias Biológicas, Instituto Politécnico Nacional, Mexico City, Mexico; 5 Central de Microscopia, Escuela Nacional de Ciencias Biológicas, Instituto Politécnico Nacional, Mexico City, Mexico; 6 Laboratorio de Investigación de Materiales Dentales y Biomateriales, Departamento de Estudios de Posgrado e Investigación, Universidad Nacional Autónoma de México, Mexico City, Mexico

**Keywords:** Primary explant culture, Thymus regeneration, Clonogenic thymic epithelial cells, Adult stem cells, Regenerative medicine

## Abstract

**Background:**

Thymic epithelial cells (TECs) are responsible for shaping the repertoires of T cells, where their postnatal regeneration depends on a subset of clonogenic TECs. Despite the implications for regenerative medicine, their cultivation and expansion remain challenging. Primary explant cell culture is a technique that allows the seeding and expansion of difficult-to-culture cells. Here, we report a reliable and simple culture system to obtain functional TECs and thymic interstitial cells (TICs).

**Methods:**

To establish primary thymic explants, we harvested 1 mm cleaned fragments of thymus from 5-week-old C57/BL6 mice. Tissue fragments of a complete thymic lobe were placed in the center of a Petri dish with 1 mL of DMEM/F-12 medium supplemented with 20% fetal bovine serum (FBS) and 1% penicillin‒streptomycin. To compare, thymic explants were also cultivated by using serum-free DMEM/F-12 medium supplemented with 10% KnockOut™.

**Results:**

We obtained high numbers of functional clonogenic TECs and TICs from primary thymic explants cultivated with DMEM/F-12 with 20% FBS. These cells exhibited a highly proliferative and migration profile and were able to constitute thymospheres. Furthermore, all the subtypes of medullary TECs were identified in this system. They express functional markers to shape T-cell and type 2 innate lymphoid cells repertoires, such as Aire, IL25, CCL21 and CD80. Finally, we also found that ≥ 70% of lineage negative TICs expressed high amounts of Aire and IL25.

**Conclusion:**

Thymic explants are an efficient method to obtain functional clonogenic TECs, all mTEC subsets and different TICs Aire^+^IL25^+^ with high regenerative capacity.

**Supplementary Information:**

The online version contains supplementary material available at 10.1186/s13287-023-03529-8.

## Introduction

Thymic epithelial cells (TECs) regeneration has become of great interest for its potential to re-educate entire repertoires of T cells for treating immunodeficiencies and for inducing allotransplant and xenotransplant tolerance [1–4]. Several groups have proposed the existence of adult thymic epithelial stem cells (TESCs) or thymic epithelial progenitor cells (TEPCs) to explain the regeneration model of the postnatal thymus [5–7]. However, there are still some controversies in defining their specific phenotype and stemness features [8]. Recently, various groups have performed single-cell RNA-seq analysis of the thymic epithelium compartments, being not able to confirm the existence of a TEPC signature. Instead of this, a subset of transit-amplifying thymic epithelial cells (TAC-TECs) or clonogenic TECs has been found [9, 10]. These cells do not express a signature with specific stem cell markers and do not have classic features of adult stem cells (ASCs), but they possess a specific signature related to cell cycle progression, proliferation and chromatin remodelling. One of the most important problems in the study of TAC-TECs or clonogenic TECs is the difficulty in expanding them in vitro [11]. Regenerative medicine offers distinct methodologies that could solve this problem. Primary explant culture is a technique that have allowed the expansion of epithelial cells in several models without using enzymatic reagents and irradiated feeder layer cells [12–15]. Therefore, the objective of this work was to design an easier and efficient methodology using primary thymic explant cultures to obtain clonogenic TECs and thymic interstitial cells (TICs) with high regenerative capacity.

## Materials and methods

### Thymic explant cultures

Thymi were obtained from 5-week-old male C57BL/6 mice. All animal experiments were approved by the Internal Committee for the Care and Use of Laboratory Animals (CICUAL) of the National Autonomous University of Mexico (UNAM) (CICUAL-FO/0011/2022). Mice were euthanized using carbon dioxide to harvest the thymus. To perform thymic explant cultures, we cleaned thymi of debris and we removed cells from the fraction 1 and 2. The protocol for cleaning thymic fragments is shown is the Additional file 1: Figs S1, 2 and Table S1. Cleaned thymic fragments from individual lobes were placed in the center of a Petri dish 7 × 7 cm with 1 mL of DMEM/F-12 medium supplemented with 20% foetal bovine serum (FBS) (Gibco™ 16250078) and 1% penicillin‒streptomycin (Gibco™ 15140122), always leaving an air phase to promote cell proliferation and migration. It is important to mention that a supplementation with 10% of FBS does not enrich epithelium, only CD45^−^EpCAM^−^ TICs. Thymic explants from the control group were cultivated with serum-free medium supplemented with 10% KnockOut™. At 12 h, we removed the medium and carried out three washes with sterile 1 × PBS to remove remaining CD45^+^ TICs that could also inhibit the growth of clonogenic TECs and TICs (Fraction 3 cells) (Additional file 1: Fig. S3). Then, we filled up the Petri dish to 2 mL, and we changed the medium every 5 days. Tissue fragments were removed at day 10 to guarantee the growth of epithelium. A flowchart and a video of the entire methodology are shown in the Additional file 1: Fig. S4, and the characterization of the obtained fraction 2 TICs is shown in Additional file 1: Figs. S5, 6.

### Hanging-drop generation system for thymospheres

Explant derived cells were detached using prewarmed trypsin with 2.5% EDTA for 10 min and cell scraper (because epithelial cells are highly attached to the plate). We adjusted 2 × 10^3^ cells in 25 µL of DMEM/F-12 supplemented medium. We dispensed 40 drops of 25 µL onto the inner surface of the Petri lid 7 × 7. Then, with a smooth movement, we twisted the lid to leave the drops hanging. Finally, we collocated 6 mL of sterile 1 × PBS in the container to prevent the drops from drying up and left under standard culture conditions. Photos were taken at days 5 and 10.

### FACS, bioinformatics analysis and functional proliferation essay

FACS analysis was performed in the BD Influx Cell Sorter, and data analysis was done using the FlowJo™ v10.8.1 software package. We made FMO controls for each analyzed marker. Staining buffer was prepared with 1% bovine serum albumin (BSA) and cell Permeabilization Buffer with 1% BSA and 0.30% Triton ™ X-100 (Cat. 39487). All samples were firstly incubated with 0.5 µL of TruStain FcX™ (CD16/32 anti-mouse) (BioLegend® 101319) for 20 min to superblock the fragment crystallizable receptors (FcRs) before the staining of surface markers. The analyzed markers were: Aire eFluor™ 660 (Cat. 50593480), CD326 Brilliant Violet™ 510 (1191155), CD45 Alexa Fluor®700 (1386045), CD11b PE (553311), IL-17E/IL-25 Alexa Fluor® (IC13991S-100UG) and Podoplanin PE/Dazzle™594 (1237095). To block the fragment, crystallizable receptors (FcRs) present in organelles and vesicles of the secretory pathway and to avoid nonspecific intracellular staining samples were also incubated with TruStain FcX™ after cell permeabilization and before staining of IL25 (Additional file 1: Fig. S7a-d). The proliferation assay was performed with CFSE BioLegend® (423801) cell division tracker at 5 mM on day 5 when a considerable number of cells emerged from the explants. We incubated the cells with CFSE for 5 days before analysis. Cells were trypsinized for 3 min without using a cell scraper to separate TICs, which are out of the epithelial cell colonies and were stained for FACS.

### Immunofluorescences

Functionalized 5 × 6 mm coverslips with bovine gelatin at 1% were sterilized using autoclave and were placed in 24-well plates to seed explants. At day 5, samples were processed on the well for immunofluorescence. The background unspecific fluorescence was turned off with a buffer of NH_4_Cl at 0.25%. Cytometry antibodies were used as primary antibodies. Their dilutions are shown in Additional file 1: Table S2. The secondary Goat anti-Rat IgG (H + L) Cross-Adsorbed Secondary Antibody, Alexa Fluor™ 568 (Cat. A-11077) was utilized at 1:500 dilution. Both primary and secondary antibodies were incubated for 2 h.

## Results

**Fig. 1:**
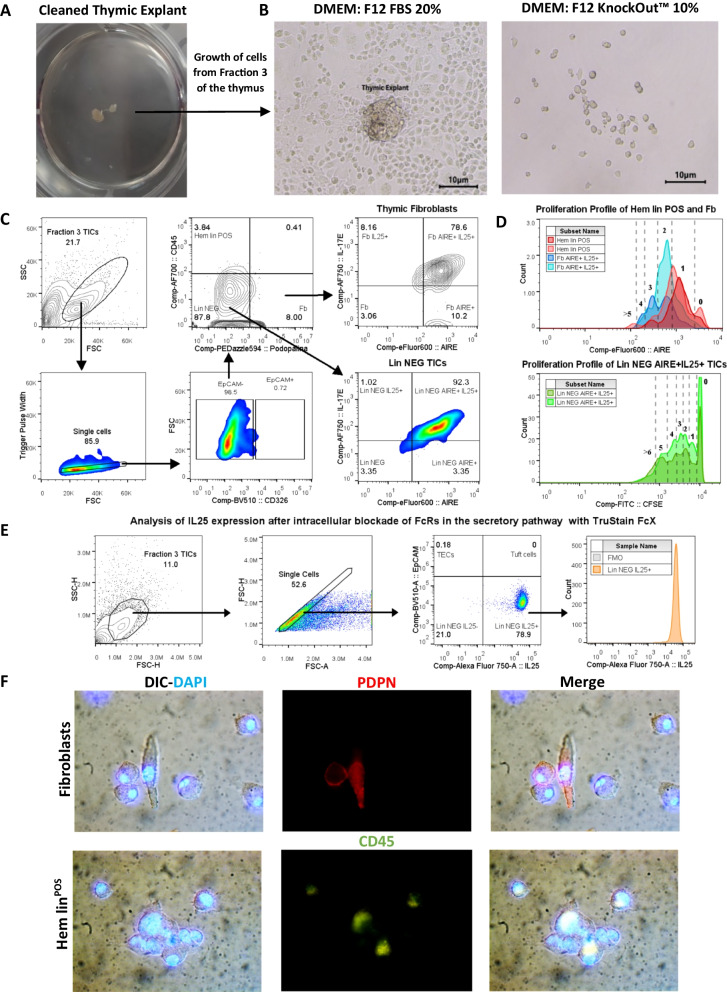
The growth of clonogenic TECs in the explant niche is supported by TICs including thymic fibroblasts, hematopoietic cells, and lineage negative cells that express high amounts of Aire and IL25 (IL-17E).** A** Cells from Fraction 3, which include clonogenic TECs and TICs, derived from cleaned thymic explants. **B** Representative images of epithelial cell colonies derived from thymic explants cultured with DMEM/F-12 FBS 20% and with DMEM/F-12 KnockOut™ 10% at day 14. The control group with DMEM/F-12 KnockOut™ 10% only exhibited small colonies of epithelial cells. Scale bar: 10 µm *D* = 10 *n* = 5. **C** Gating strategy to characterize TICs derived from fraction 3, including thymic fibroblasts, cells from the hematopoietic lineage (Hem lin^POS^) and lineage negative cells (lin^NEG^). Both lin^NEG^ cells and fibroblasts highly express IL25 (IL17E) and Aire (*n* = 2). **D** Proliferation profile of TICs derived from thymic explants. Blue histograms represent the proliferation profile of thymic fibroblasts, red histograms represent the proliferation profile of Hem lin^POS^ cells and green histograms represent the proliferation profile of lin^NEG^ cells (*n* = 2). **E** Intracellular blockade of FcRs in organelles and vesicles of the secretory pathway with Trustain FcX™ decreases the percentage of lin^NEG^ IL25^+^ TICs by 15%, but there are still more than 75% of lin^NEG^ TICs with truly positive expression of IL25 (*n* = 2). **F** Thymic fibroblasts and Hem lin^POS^ cells, interact and support the growth of clonogenic TECs in explant niche. Scale bar: 20 µm *D* = 100 *n* = 2. DIC = Differential interference contrast, Fb = Fibroblasts, FMO = Fluorescence minus one, FcRs = Fragment Crystallizable Receptors, Hem lin^POS^ = Hematopoietic lineage positive cells, lin^NEG^ = Lineage negative cells, PDP* n* = Podoplanin, TICs = Thymic Interstitial Cells

**Fig. 2:**
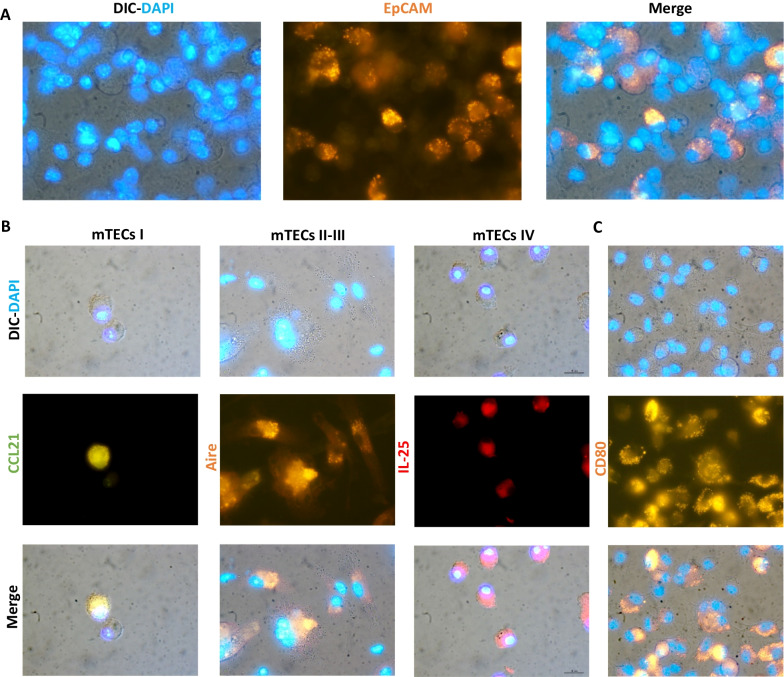
Clonogenic TECs derived from thymic explants restore the four subsets of medullary TECs and most of these cells highly express CD80. **A** Most cells derived from primary explants cultured in functionalized coverslips express the classic epithelial marker EpCAM (CD326). **B** The four subsets of medullary TECs were present in the epithelial cell colonies derived from thymic explants. **C** Most cells derived from explants highly express CD80. **A**–**C** scale bar: 20 µm *D* = 100 *n* = 2. DIC = differential interference contrast,  mTECs = medullary thymic epithelial cells

**Fig. 3:**
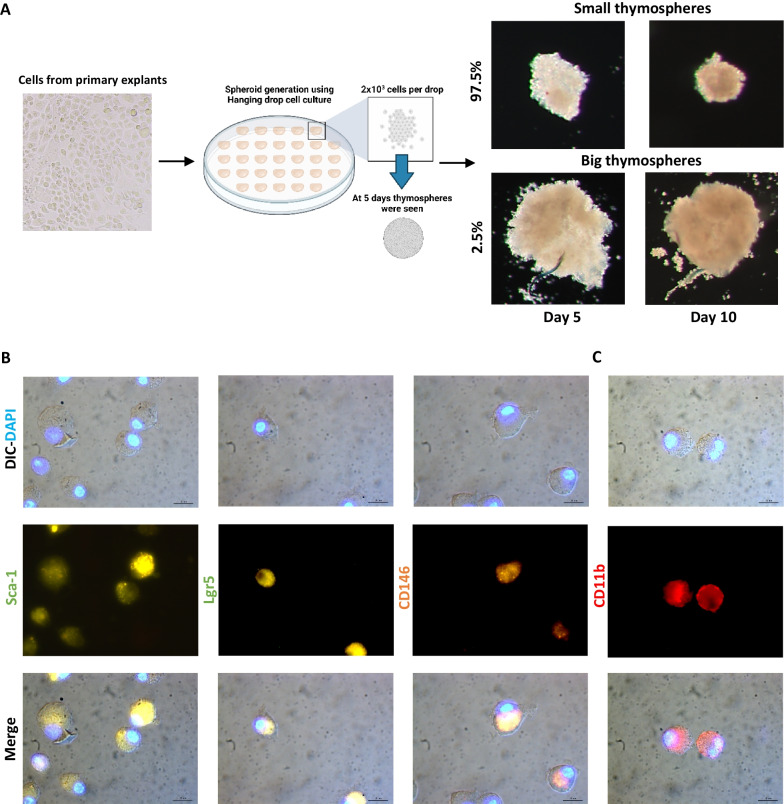
Cells derived from thymic explants can constitute thymospheres and present heterogeneous expression of stem cell markers.** A** Cells from primary explants were cultured using a hanging-drop generation system and constituted small thymospheres in 97.5% and large thymospheres in 2.5% of cases. **B** Different epithelial cells express the stem cell markers Sca-1, Lgr5 and CD146. **C** Various epithelial cells derived from explants express the integrin CD11b. **B**, **C** scale bar: 20 µm *D* = 100 *n* = 2. DIC = differential interference contrast

First, we compared the colony-forming cells capacity of thymic primary explants under two different conditions. Particularly, we observed the feasibility to obtain large epithelial cell colonies by cultivating thymic explants with DMEM/F-12 medium supplemented with FBS at 20% (Fig. 1A, B). In contrast, we observed that explants cultivated with 10% KnockOut™ gave only small colonies with few numbers of epithelioid cells (Fig. 1B). Beyond epithelioid cells, different TICs derived from thymic explants. We identified thymic fibroblasts, hematopoietic cells (Hem lin^POS^), and Aire^+^ IL-25(IL17-E)^+^ lineage negative (lin^NEG^) cells (non-hematopoietic, epithelial or fibroblast cells) in the niche of the explant (Fig. 1C). We also found that these cells undergo multiple cell division cycles within the niche of the explant (Fig. 1D). To test whether IL25 expression was not a consequence of nonspecific staining mediated by FcRs associated with organelles and vesicles of the secretory pathway, we performed additional blocking with anti-CD16/CD32 antibodies after cell permeabilization. With this, we observed that the percentage of IL25^+^ TICs decreased by 15%. However, we found that 75% of TICs really expressed IL25 (Fig. 1E). Using immunofluorescence analysis, we found that epithelioid cells strongly interact with fibroblasts, Hem lin^POS^ cells and the Aire^+^IL-25^+^ lin^NEG^ TICs. This suggests these cells could potentially act as feeder cells that favor the growth of clonogenic TECs by secreting multiple growth factors and cytokines (Fig. 1F) [16]. This would explain why clonogenic TECs, which are difficult-to-cultivate cells, can easily grow in the explant niche. Next, we performed immunofluorescence staining to further corroborate the identity of cell colonies with epithelioid morphology. Our results identified most of the cells derived from cell colonies expressed the classic epithelial marker EpCAM (CD326) (Fig. 2A). Likewise, we identified the four subsets of medullary TECs in the epithelial cell colonies. Specifically, we found mTECs I CCL21^+^, mTECs II Aire^hi^, mTECs III Aire^lo^ and mTECs IV or Tuft cells IL25^+^ (Fig. 2B). These findings are relevant as all of these subsets are responsible for performing the negative selection for lymphopoiesis and, in the case of mTEC IV, shaping the repertoires of type 2 innate lymphoid cells (ILC2s) [17]. Moreover, we found that most cells express the co-stimulatory molecule ligand CD80, indicating they could have a high antigen-presenting capacity (Fig. 2C) [18]. Finally, clonogenic TECs derived from explants exhibited a high capacity to constitute thymospheres (Fig. 3A) and present a heterogeneous expression of stem cell markers, such as Sca-1, Lgr5 and CD146 (Fig. 3B) [19, 20]. These markers enhance the function of stem cells from other organs. For example, Mesenchymal stem cells (MSCs) that express CD146 have superior cell properties such as greater proliferation, differentiation, migration and immune regulation abilities [21]. Moreover, beyond the heterogenous expression of stem cells markers, the capacity to constitute thermospheres represents a functional marker of a certain degree of stemness. Finally, these cells also exhibited high expression of the integrin CD11b. This result interesting due to some authors have unexpectedly found that CD11b is expressed in several epithelial ovarian cancer (EOC) lines and has been associated with a high regenerative capacity and cell resistant treatment against chemotherapy (Fig. 3C) [22].

## Discussion

Here, we present a simpler, efficient and fast method to obtain clonogenic TECs and Aire^+^IL25^+^ TICs with high regenerative capacity. To emphasize in the simplicity and efficiency of our method is important to recapitulate others already tested by different groups. In brief, Villegas et al. expanded human clonogenic TECs with medullary phenotype from human thymic explants. These cells expressed claudin 4 and cytokeratins 5/14.They used RPMI-1640 medium supplemented with 20% horse serum, Ultroser G, l-glutamine and penicillin/streptomycin [23]. Likewise, Sekai et al. also succeeded in expanding mouse clonogenic TECs. They called these cells mTEC stem cells (mTESCs). mTESCs exhibited a high expression of SSEA-1^+^ Cld 3, 4^hi^ [11, 24]. They obtained these cells from thymi of mice younger than 4 weeks, using enzymatic digestion and additional supplements, such as epithelial growth factor (EGF) and LIF. Moreover, Campnoti et al. successfully expanded postnatal clonogenic TECs CD49f^+^ from human thymi of cortical and medullary origin over 3T3-J2 feeder fibroblasts and using cFAD medium (3:1 of DMEM-1X and F-12 Nut Mix) supplemented with 10% FBS, 1% penicillin and streptomycin, Hydrocortisone, Cholera Toxin, Triiodothyronine and Insulin. They also added human epithelial growth factor (hEGF) at day 3 [9]. In our case, we only use DMEM/F-12 basal medium supplemented with 20% of FBS that was changed every 5 days, obtaining clonogenic TECs and TICs from thymi of 5-week-old mice in an average of 5 days. The comparison of our methodology with these methodologies is shown in the Additional file 1: Table. S3.

Following this simple methodology, we obtained clonogenic TECs and TICs from postnatal thymi, avoiding the use of enzymatic procedures, growth factors and irradiated feeder cells. Apparently, the success in expanding clonogenic TECs from thymic explants lies in the interaction with different subtypes of TICs. Among these cells are small fractions of fibroblasts, Hem lin^POS^ cells and a large number of different types of lin^NEG^ TICs that express Aire and IL25. Because these cells secrete a high number of cytokines, growth factors and hormones, we have been able to avoid the use of growth factors and irradiated feeder cells. Unexpectedly, we found these TICs really express high amounts of IL25 (Fig. 1 E and Additional file 1: Fig. S7), which is interesting as this has not been previously reported [25]. The high expression of IL25 could even be associated with the high regeneration of the thymic epithelium in the explant's niche, but future studies will be necessary to confirm it. Finally, single-cell sequencing studies at the transcriptomic level have not been able to find a potential TEPC signature that differentiates them from mature TECs [10]. In this study, we found that beyond the expression of stem cell markers, the distinctive features of our clonogenic TECs are their great potential for expansion and regeneration, as well as their ability to constitute thymospheres. Thus, it is possible that clonogenic TECs obtained by Sekai et al., Bonfanti et al., Villegas et al. and those obtained in this study could be the same.

## Conclusions

This work demonstrates that primary thymic explants are an efficient technique to obtain great number of clonogenic TECs and TICs with a great potential to regenerate functional thymi to treat immunodeficiencies.

### Supplementary Information

**Additional file 1.** Supplementary figures and tables.

## Data Availability

The datasets used and analyzed during the current study are available from the corresponding author on reasonable request.

## Abbreviations

ASCs: Adult stem cells
DIC: Differential interference contrast
EGF: Epithelial growth factor
EOC: Epithelial ovarian cancer
EpCAM: Epithelial cell adhesion molecule
FBS: Fetal bovine serum
FCRs: Fragment crystallizable receptors
FMO: Fluorescence minus one
hEGF: Human epithelial growth factor
Hem lin^POS^: Hematopoietic lineage cells
ILC2s: Innate lymphoid cells type 2
mTECs: Medullary thymic epithelial cells
mTESCs: Medullary thymic stem cells
MSCs: Mesenchymal stem cells
lin^NEG^: Lineage negative cells
PDPN: Podoplanin
TAC-TECs: Transit-amplifying thymic epithelial cells
TECs: Thymic epithelial cells
TEPCs: Thymic epithelial progenitor cells
TESCs: Thymic epithelial stem cells
TICs: Thymic interstitial cells
