# Correction: Primary explants of the postnatal thymus allow the expansion of clonogenic thymic epithelial cells that constitute thymospheres

**DOI:** 10.1186/s13287-024-03665-9

**Published:** 2024-02-14

**Authors:** Juan M. Ocampo-Godinez, Jose L. Gonzalez-Quiroz, Hector Cote-Palafox, Elizabeth George, Jael A. Vergara-Lope Nuñez, Guillermo Villagomez-Olea, Febe C. Vazquez-Vazquez, Edgar O. Lopez-Villegas, Gloria Leon-Avila, Maria L. Dominguez-Lopez, Marco A. Alvarez-Perez

**Affiliations:** 1 Laboratorio de Bioingeniería de Tejidos, División de Estudios de Posgrado E Investigación, Universidad Nacional Autónoma de Mexico, Mexico City, Mexico; 2 Laboratorio de Genética, Departamento de Zoología, Escuela Nacional de Ciencias Biológicas, Instituto Politécnico Nacional, Mexico City, Mexico; 3 Carrera de Médico Cirujano, Facultad de Estudios Superiores Iztacala, Universidad Nacional Autónoma de Mexico, Mexico City, Estado de Mexico Mexico; 4 Laboratorio de Inmunoquímica I, Departamento de Inmunología, Escuela Nacional de Ciencias Biológicas, Instituto Politécnico Nacional, Mexico City, Mexico; 5 Central de Microscopia, Escuela Nacional de Ciencias Biológicas, Instituto Politécnico Nacional, Mexico City, Mexico; 6 Laboratorio de Investigación de Materiales Dentales y Biomateriales, Departamento de Estudios de Posgrado E Investigación, Universidad Nacional Autónoma de México, Mexico City, Mexico

**Correction: Stem Cell Research & Therapy (2023) 14:312** 10.1186/s13287-023-03529-8

The original article contains an erroneous attribution of co-author, Gloria Leon-Avila to affiliation #3. Dr Leon-Avila should instead be affiliated to affiliation #2 as shown in this correction article.

