# Supplementary material for: Primary explants of the postnatal thymus allow the expansion of clonogenic thymic epithelial cells that constitute thymospheres
Source: Stem Cell Res Ther. 2023 Oct 31;14:312. doi: 10.1186/s13287-023-03529-8 (PMC10617125; doi:10.1186/s13287-023-03529-8)
Supplement: Supplementary file 1 — Additional file 1. Supplementary figures and tables. [file 13287_2023_3529_MOESM1_ESM.docx]

**Supplementary**

**Protocol and validation of the methodology for washing thymic fragments**

Thymi were cut into 1 mm fragments and gently squeezed with the plunger of a syringe. Then, squeezed fragments were rinsed thrice with sterile 1x PBS. This makes it possible to eliminate up to ~90 x 10^6^ cells from fraction 1 of the thymus, which is mainly constituted of thymocytes and recirculating lymphocytes (Fig. S1 a, b). The Petri dish was tilted at 45° to allow the 1x PBS to run down, and the thymic fragments were joined with the help of the pipette tip or a cell scrapper to be sucked and transferred to a centrifuge tube with 12 mL of sterile 1x PBS.

| **Sample** | **Number of cells obtained from Fraction 1** |  |
| --- | --- | --- |
|  |  |  |
| **Lobe 1** | 20 000 000 |  |
| **Lobe 2** | 49 375 000 |  |
| **Lobe 3** | 70 000 000 |  |
| **Lobe 4** | 41 250 000 |  |
| **Average per Thymic Lobe** | 45 156 250 |  |
| **Average per Thymus** | 90 312 500 |  |

**
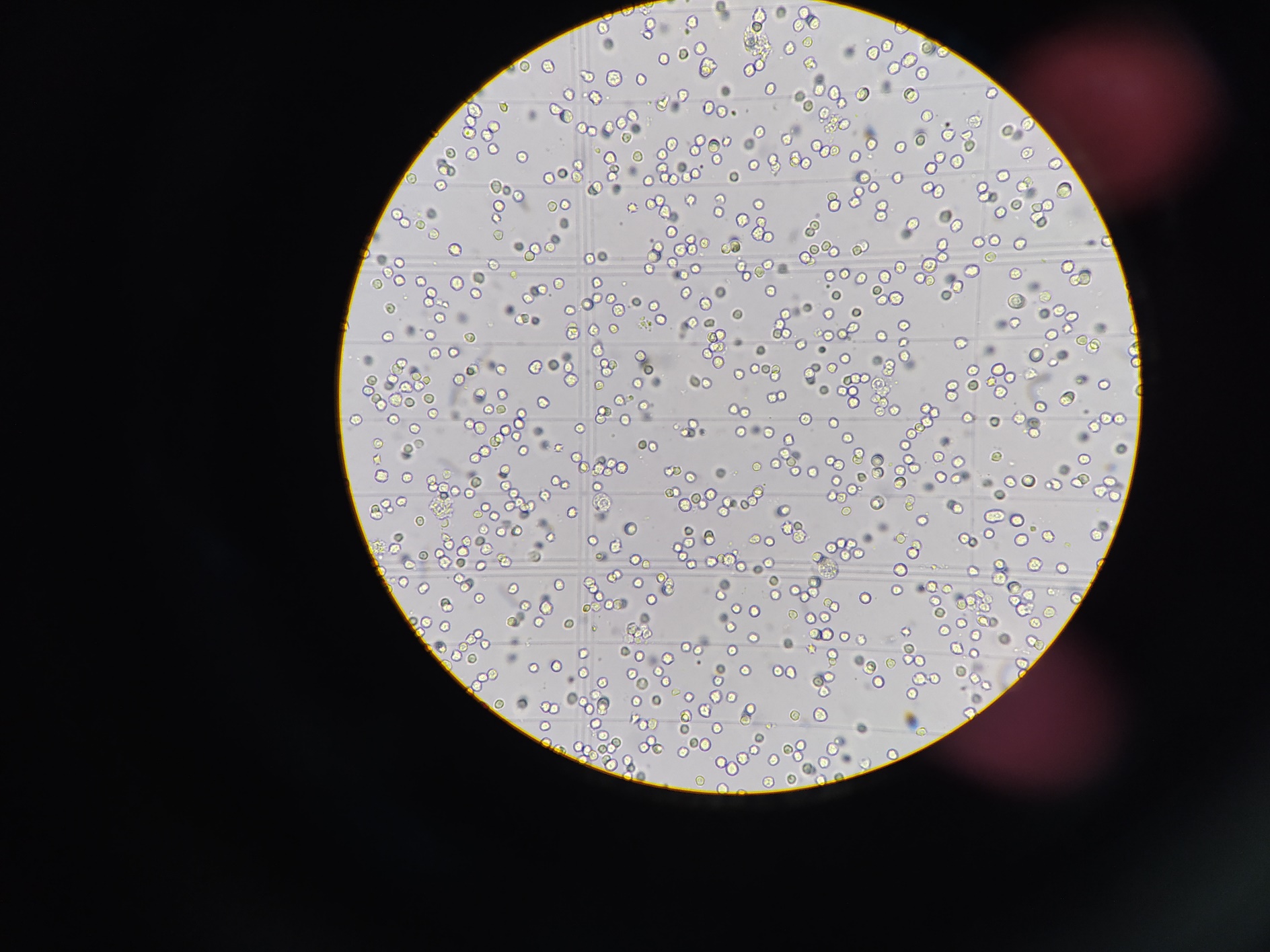
**

**B**

**A**

**Supplementary Figure S1. Reproducibility of the mechanical removal of thymic debris and cells from fraction 1 of the postnatal thymus. A** Representative image of cells obtained from postnatal thymus fraction 1 after three washes of squeezed thymic fragments. Most of these cells have a lymphoid morphology and correspond to thymocytes and recirculating lymphocytes (n=4). **B** Number of thymic fraction 1 cells extracted from each thymic lobe after three washes of the squeezed fragments. Each lobe was processed as an individual sample (n=4) by two researchers not previously trained in the methodology to evaluate its reproducibility.

After removing cells from fraction 1 of the thymus, cells belonging of fraction 2 were removed. For this, fast circular hand-stirring movements were performed 10 times in one direction and 10 times in the opposite direction. Then, we perform vigorous shaking from top to bottom 10 times. These steps complete a cycle and 10 cycles were repeated in total. Subsequently, to settle thymic fragments, they were centrifuged at 200 xg for 2 min at room temperature. The obtained supernatant was placed in another 15 mL tube and centrifuged at 550 xg at room temperature for 5 minutes to obtain a pellet of TICs. The 1x PBS was decanted once again in the first 15 mL tube that contained the thymic fragments. This wash cycles were repeated 5 times and up to ~1 million TICs were allowed to be removed (Fig. S2a, b and Table S1). The cells obtained from cycle washes were called TICs of fraction 2 of the thymus. A flowchart with a video of the entire methodology is shown in the Fig. S4 and the characterization of cells obtained after washes is shown in Figs. S5, 6.

**
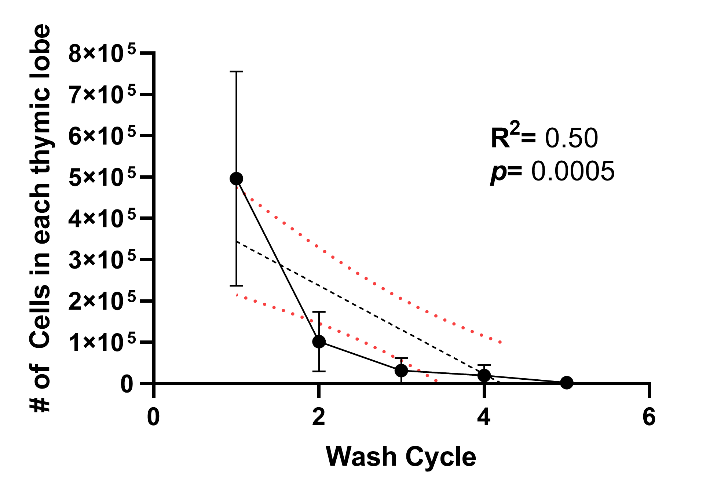
**
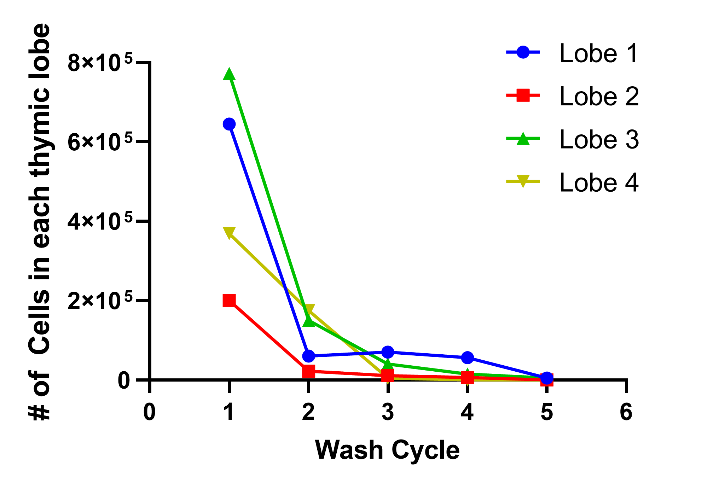


**A**

**B**

**C**

**Supplementary Figure S2. Reproducibility of the mechanical removal of cells from the fraction 2 of the postnatal thymus. A** Scatter plot with smooth lines representing the collection of cells from thymus fraction 2 after each washing cycle. **B** Linear regression analysis predicted that the number of thymic fraction 2 TICs removed from thymic tissue depends on the number of wash cycles performed. The bars represent the SD. Red dashed lines indicate 95% confidence intervals (CI). The linear regression equation was Y=-107063*X+451563. The number of X values were 20 and the number of Y replicates were 4. R2= 0.50 and p= 0.0005. For these experiments each lobule was processed as an individual sample (n=4) by two researchers not previously trained in the methodology.

**
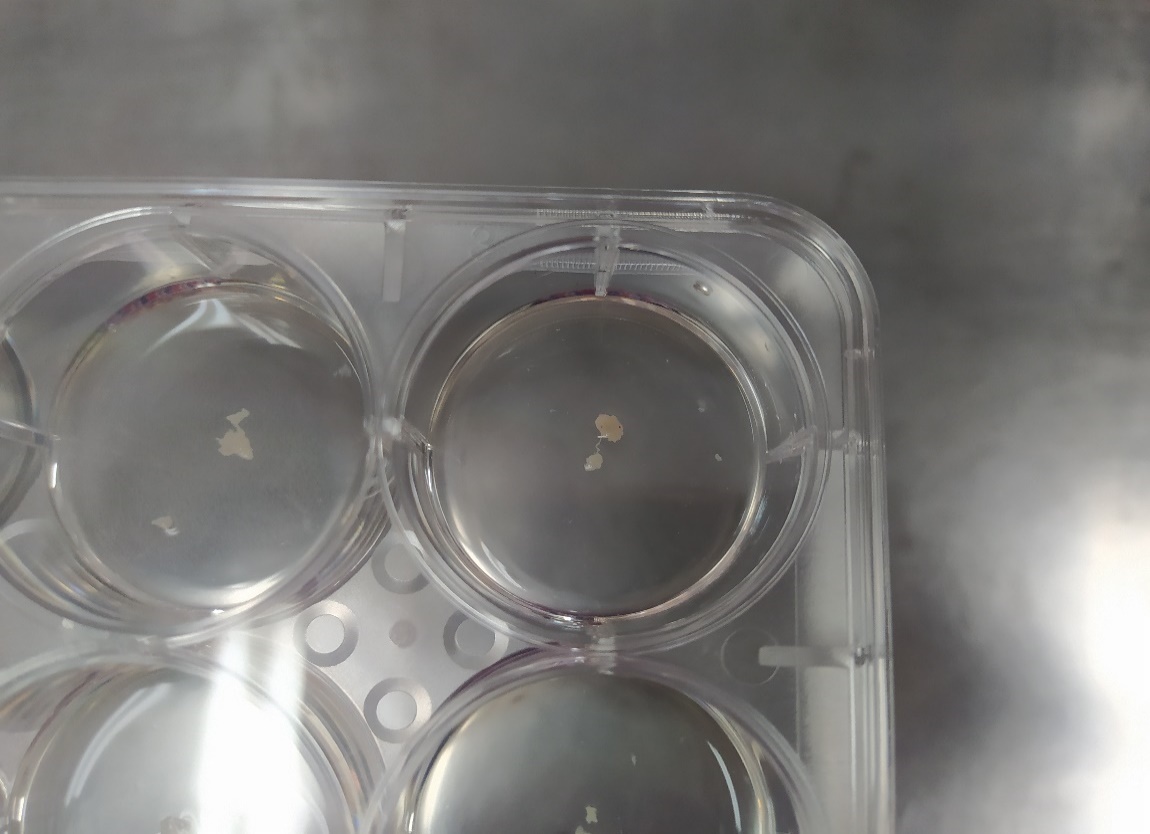

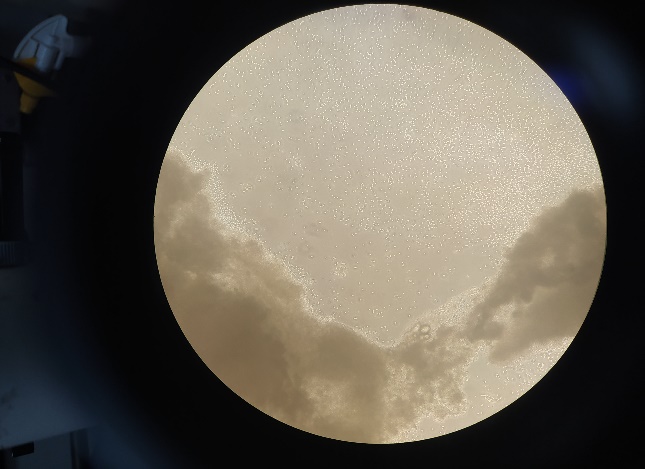
**

**Before 3 washes**

|  | **Replicate Values** | | | | | |
| --- | --- | --- | --- | --- | --- | --- |
|  | **# of Thymic Interstitial Cells TICs from Fraction 2 of the Thymus** | | | | | |
| **Wash Cycle** | **Lobule 1** | **Lobule 2** | **Lobule 3** | **Lobule 4** | **Average per Thymic Lobule** | **Average per Thymus** |
| **1** | 645000 | 200000 | 772500 | 368750 | 496562.5 | 993125 |
| **2** | 60000 | 22500 | 150000 | 175000 | 101875 | 203750 |
| **3** | 70000 | 11250 | 40000 | 5000 | 31562.5 | 63125 |
| **4** | 56250 | 6250 | 15000 | 0 | 19375 | 38750 |
| **5** | 5000 | 0 | 5000 | 0 | 2500 | 5000 |

**A**

**After 3 washes**

|  | **Replicate Values** | | | | | |
| --- | --- | --- | --- | --- | --- | --- |
|  | **# of Thymic Interstitial Cells TICs from Fraction 2 of the Thymus** | | | | | |
| **Wash Cycle** | **Lobule 1** | **Lobule 2** | **Lobule 3** | **Lobule 4** | **Average per Thymic Lobule** | **Average per Thymus** |
| **1** | 645000 | 200000 | 772500 | 368750 | 496562.5 | 993125 |
| **2** | 60000 | 22500 | 150000 | 175000 | 101875 | 203750 |
| **3** | 70000 | 11250 | 40000 | 5000 | 31562.5 | 63125 |
| **4** | 56250 | 6250 | 15000 | 0 | 19375 | 38750 |
| **5** | 5000 | 0 | 5000 | 0 | 2500 | 5000 |

**A**

**Thymic Explant at 12 hours**

|  | **Replicate Values** | | | | | |
| --- | --- | --- | --- | --- | --- | --- |
|  | **# of Thymic Interstitial Cells TICs from Fraction 2 of the Thymus** | | | | | |
| **Wash Cycle** | **Lobule 1** | **Lobule 2** | **Lobule 3** | **Lobule 4** | **Average per Thymic Lobule** | **Average per Thymus** |
| **1** | 645000 | 200000 | 772500 | 368750 | 496562.5 | 993125 |
| **2** | 60000 | 22500 | 150000 | 175000 | 101875 | 203750 |
| **3** | 70000 | 11250 | 40000 | 5000 | 31562.5 | 63125 |
| **4** | 56250 | 6250 | 15000 | 0 | 19375 | 38750 |
| **5** | 5000 | 0 | 5000 | 0 | 2500 | 5000 |

**A**

**A**

**Remaining CD45^+^ TICs**

|  | **Replicate Values** | | | | | |
| --- | --- | --- | --- | --- | --- | --- |
|  | **# of Thymic Interstitial Cells TICs from Fraction 2 of the Thymus** | | | | | |
| **Wash Cycle** | **Lobule 1** | **Lobule 2** | **Lobule 3** | **Lobule 4** | **Average per Thymic Lobule** | **Average per Thymus** |
| **1** | 645000 | 200000 | 772500 | 368750 | 496562.5 | 993125 |
| **2** | 60000 | 22500 | 150000 | 175000 | 101875 | 203750 |
| **3** | 70000 | 11250 | 40000 | 5000 | 31562.5 | 63125 |
| **4** | 56250 | 6250 | 15000 | 0 | 19375 | 38750 |
| **5** | 5000 | 0 | 5000 | 0 | 2500 | 5000 |

**A**

**Fully cleaned thymic explants**

|  | **Replicate Values** | | | | | |
| --- | --- | --- | --- | --- | --- | --- |
|  | **# of Thymic Interstitial Cells TICs from Fraction 2 of the Thymus** | | | | | |
| **Wash Cycle** | **Lobule 1** | **Lobule 2** | **Lobule 3** | **Lobule 4** | **Average per Thymic Lobule** | **Average per Thymus** |
| **1** | 645000 | 200000 | 772500 | 368750 | 496562.5 | 993125 |
| **2** | 60000 | 22500 | 150000 | 175000 | 101875 | 203750 |
| **3** | 70000 | 11250 | 40000 | 5000 | 31562.5 | 63125 |
| **4** | 56250 | 6250 | 15000 | 0 | 19375 | 38750 |
| **5** | 5000 | 0 | 5000 | 0 | 2500 | 5000 |

**A**

**
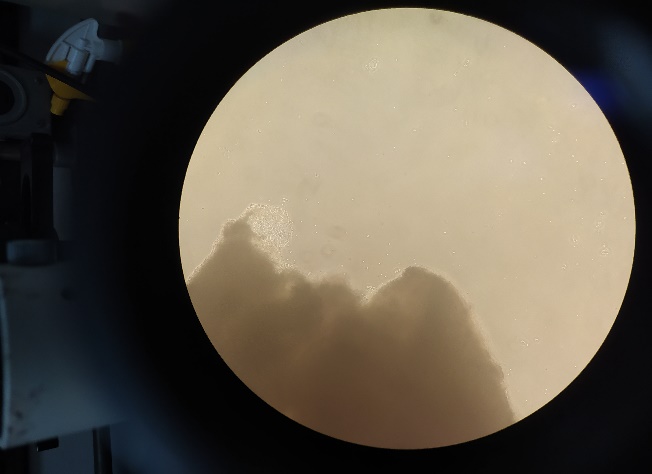
**

**Supplementary Figure S3. Additional wash at 12 hours to remove remaining CD45+ TICs. A** At 12 hours the medium was removed, and three washes were carried out with sterile 1x PBS to remove the remaining CD45^+^ TICs that inhibit the growth of cells from the fraction 3 of the postnatal thymus, which includes clonogenic TECs.

**
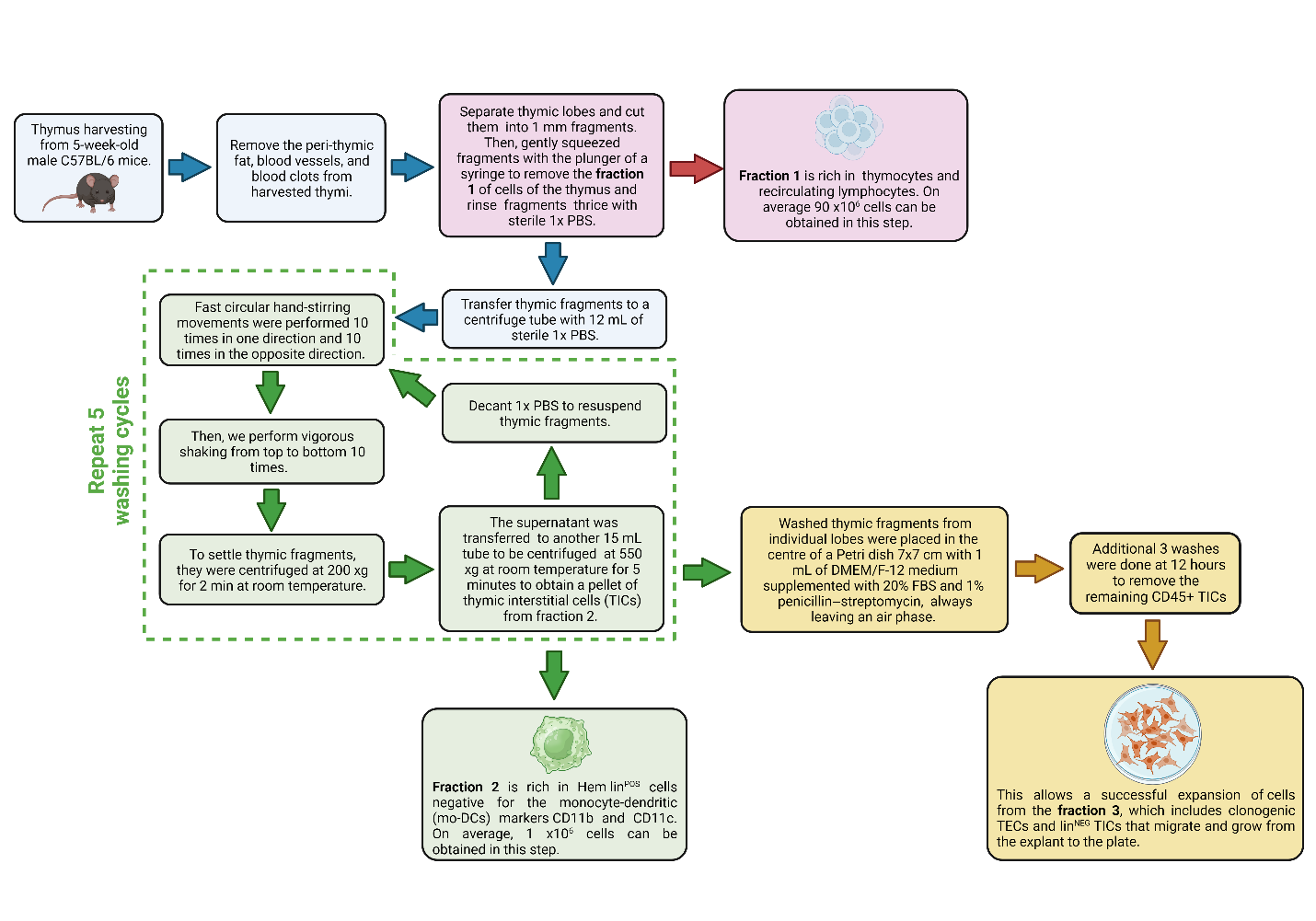
Supplementary Figure S4. Flowchart of the entire methodology.** For a better understanding of the methodology, it can be reviewed the video at the following link: <https://doi.org/10.6084/m9.figshare.24183621.v1>

Most fraction 2 TICs were Hem lin^POS^ cells negative for the monocyte-dendritic (mo-DCs) markers CD11b and CD11c ( Fig. S5). Moreover, ≤ 10% were lin^NEG^ TICs. From this lin^NEG^ TICs, 20% express the endothelial marker CD31(PECAM) and the rest were only lin^NEG^ cells (Fig. S6). Clearance of cells from fractions 1 and 2 is essential because they are in constant proliferation and consume the nutrients of the medium. This allows a successful expansion of cells from the fraction 3, which includes clonogenic TECs and lin^NEG^ TICs that migrate and grow from the explant to the plate.


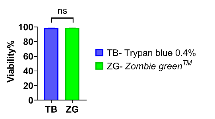
**
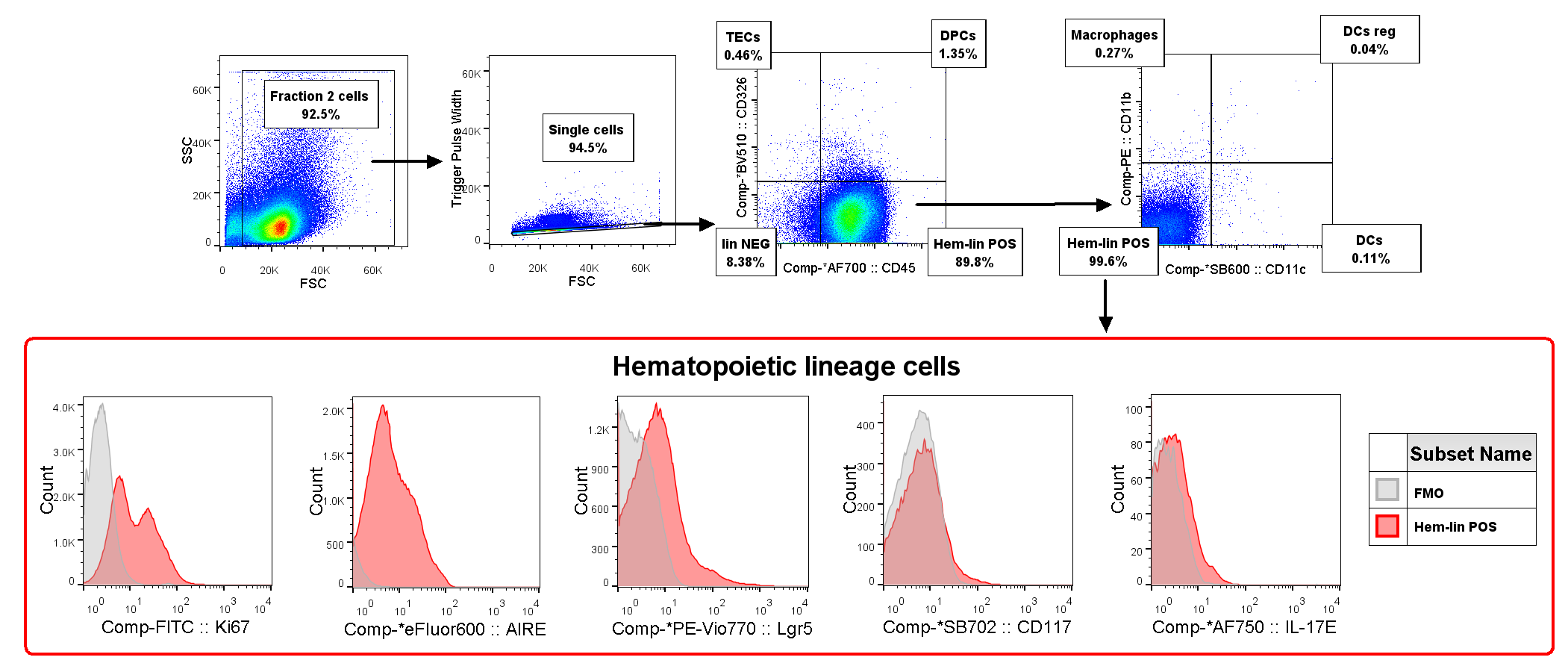
**

**C**

|  | **Replicate Values** | | | | | |
| --- | --- | --- | --- | --- | --- | --- |
|  | **# of Thymic Interstitial Cells TICs from Fraction 2 of the Thymus** | | | | | |
| **Wash Cycle** | **Lobule 1** | **Lobule 2** | **Lobule 3** | **Lobule 4** | **Average per Thymic Lobule** | **Average per Thymus** |
| **1** | 645000 | 200000 | 772500 | 368750 | 496562.5 | 993125 |
| **2** | 60000 | 22500 | 150000 | 175000 | 101875 | 203750 |
| **3** | 70000 | 11250 | 40000 | 5000 | 31562.5 | 63125 |
| **4** | 56250 | 6250 | 15000 | 0 | 19375 | 38750 |
| **5** | 5000 | 0 | 5000 | 0 | 2500 | 5000 |

**A**

**B**

|  | **Replicate Values** | | | | | |
| --- | --- | --- | --- | --- | --- | --- |
|  | **# of Thymic Interstitial Cells TICs from Fraction 2 of the Thymus** | | | | | |
| **Wash Cycle** | **Lobule 1** | **Lobule 2** | **Lobule 3** | **Lobule 4** | **Average per Thymic Lobule** | **Average per Thymus** |
| **1** | 645000 | 200000 | 772500 | 368750 | 496562.5 | 993125 |
| **2** | 60000 | 22500 | 150000 | 175000 | 101875 | 203750 |
| **3** | 70000 | 11250 | 40000 | 5000 | 31562.5 | 63125 |
| **4** | 56250 | 6250 | 15000 | 0 | 19375 | 38750 |
| **5** | 5000 | 0 | 5000 | 0 | 2500 | 5000 |

**A**

**A**

|  | **Replicate Values** | | | | | |
| --- | --- | --- | --- | --- | --- | --- |
|  | **# of Thymic Interstitial Cells TICs from Fraction 2 of the Thymus** | | | | | |
| **Wash Cycle** | **Lobule 1** | **Lobule 2** | **Lobule 3** | **Lobule 4** | **Average per Thymic Lobule** | **Average per Thymus** |
| **1** | 645000 | 200000 | 772500 | 368750 | 496562.5 | 993125 |
| **2** | 60000 | 22500 | 150000 | 175000 | 101875 | 203750 |
| **3** | 70000 | 11250 | 40000 | 5000 | 31562.5 | 63125 |
| **4** | 56250 | 6250 | 15000 | 0 | 19375 | 38750 |
| **5** | 5000 | 0 | 5000 | 0 | 2500 | 5000 |

**A**

**Supplementary Figure S5. Flow cytometry panel of the Hematopoietic cells obtained after the wash cycle steps of thymic fragments before their explant.** **A** The viability of TICs from fraction 2 before FACs analysis was ≥95%. The viability was made by trypan blue at 0.4% and also by Zombie green dye in flow cytometer obtaining a similar performance (n=4 per group). The bar represents the mean ± SEM, Shapiro‒Wilk test was used to determine normality, F test was used to determine homoscedasticity, ns = not significant. Mann‒Whitney test were used to compare groups. **B** Representative image of the gating strategy to characterize the main lineages present in the sample obtained after 5 washes (n=2). **C** Characterization of Hem-lin^POS^ cell subsets. Most of Hem-lin^POS^ cells from fraction 2 were negative for the monocyte-dendritic (mo-DCs) markers CD11b and CD11c. **D** Hem-lin^POS^ cells highly express the classic proliferation marker ki67 and the gene promiscuous marker Aire. They also express low amounts of Lgr5 and very low amounts of c-kit (CD-117) and IL25 (IL-17E). These are representative images of 2 experiments independently performed by 2 different researchers not previously trained for the methodology. DCs= Dendritic cells, DCs reg= Regulatory dendritic cells, DPCs= Dual Positive Cells, FMO= Fluorescence minus one, Hem-lin^POS^= Hematopoietic lineage positive cells, lin^NEG^= Lineage negative cells, TECs=Thymic Epithelial Cells.


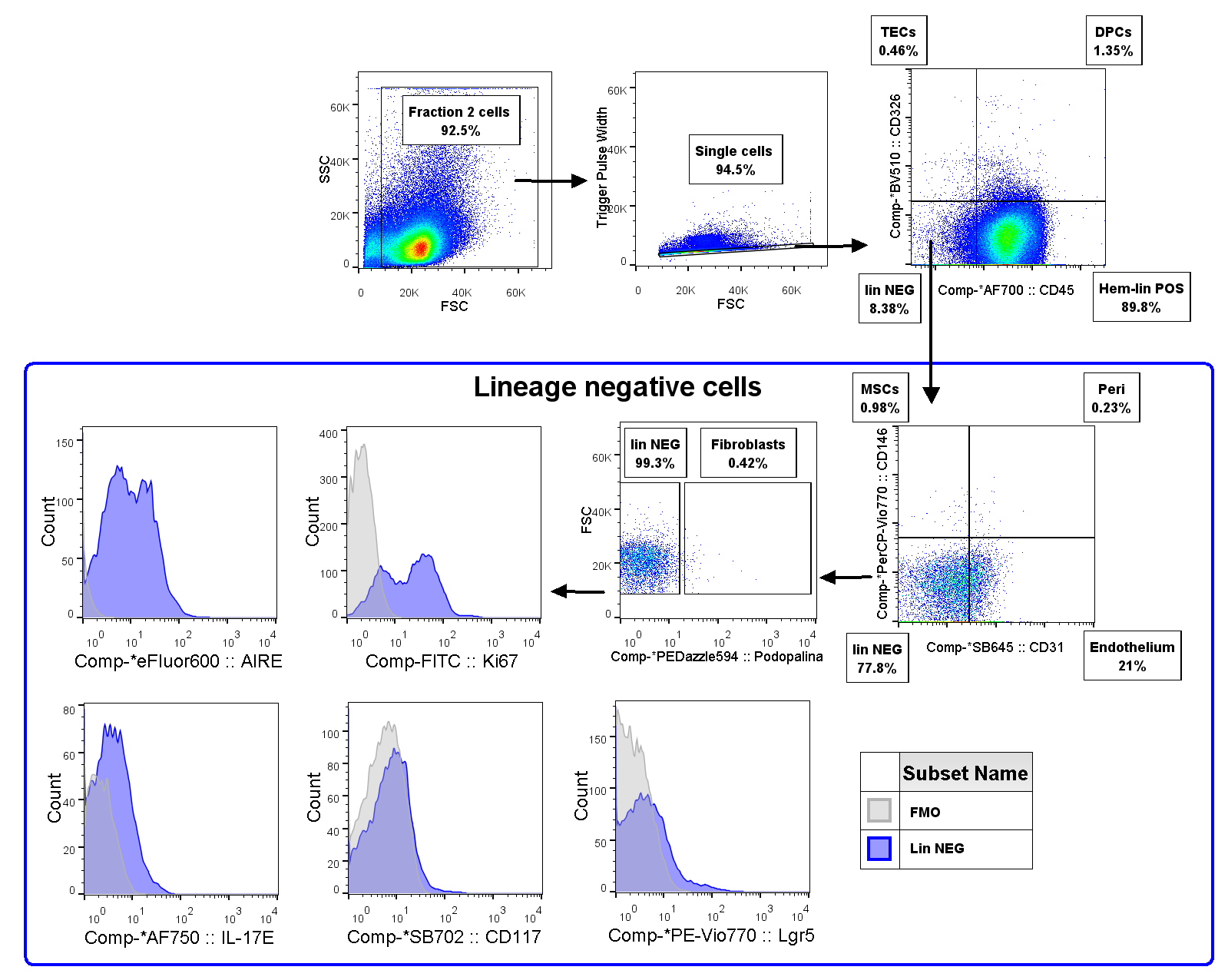
**Supplementary Figure S6. Flow cytometry panel of the lineage negative cells obtained after the different centrifugation steps of thymic fragment before their explant. A** Gating strategy to characterize the main lineages present in the sample obtained after the 5 washes (n=2). **B** Characterization of lin^NEG^ cell subsets, where 21% expressed the endothelial marker CD31, but most of them were negative for the markers of pericytes, mesenchymal stem cells (MSCs) and fibroblasts. **C** These Hem-lin^POS^ cells highly express the classic proliferation marker ki67 and the gene promiscuous marker Aire. They express low amounts of IL25 and were almost negative for Lgr5 and c-kit (CD-117). These are representative images of 3 experiments independently performed by 3 different researchers. DPCs= Dual Positive Cells, FMO= Fluorescence minus one, Hem-lin^POS^= Hematopoietic lineage positive cells, lin^NEG^= Lineage negative cells, MSCs=Mesenchymal Stem Cells, Peri= Pericytes, TECs=Thymic Epithelial Cells.

**C**

**B**

**A**


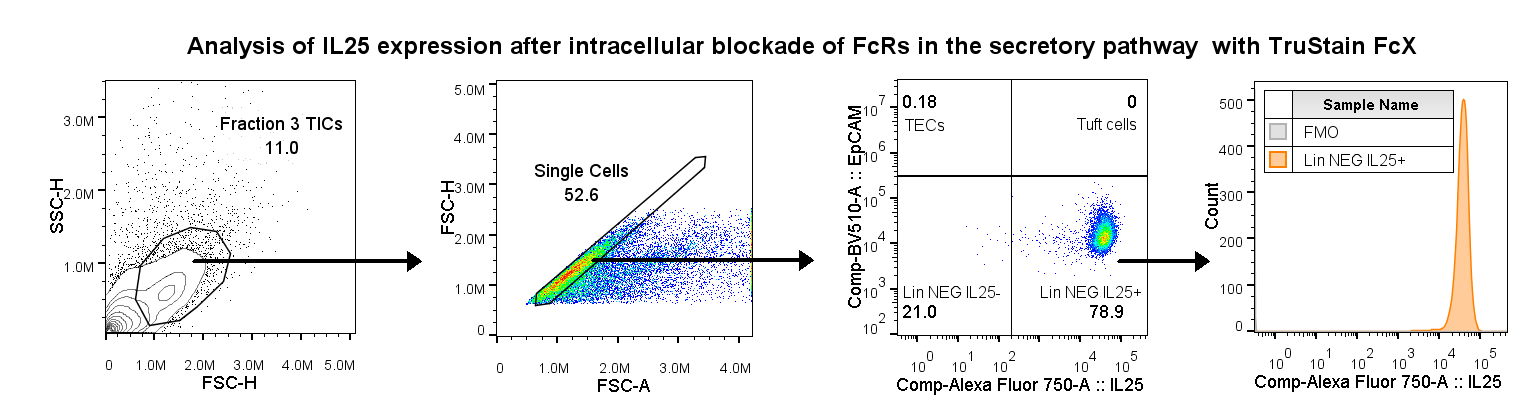


**
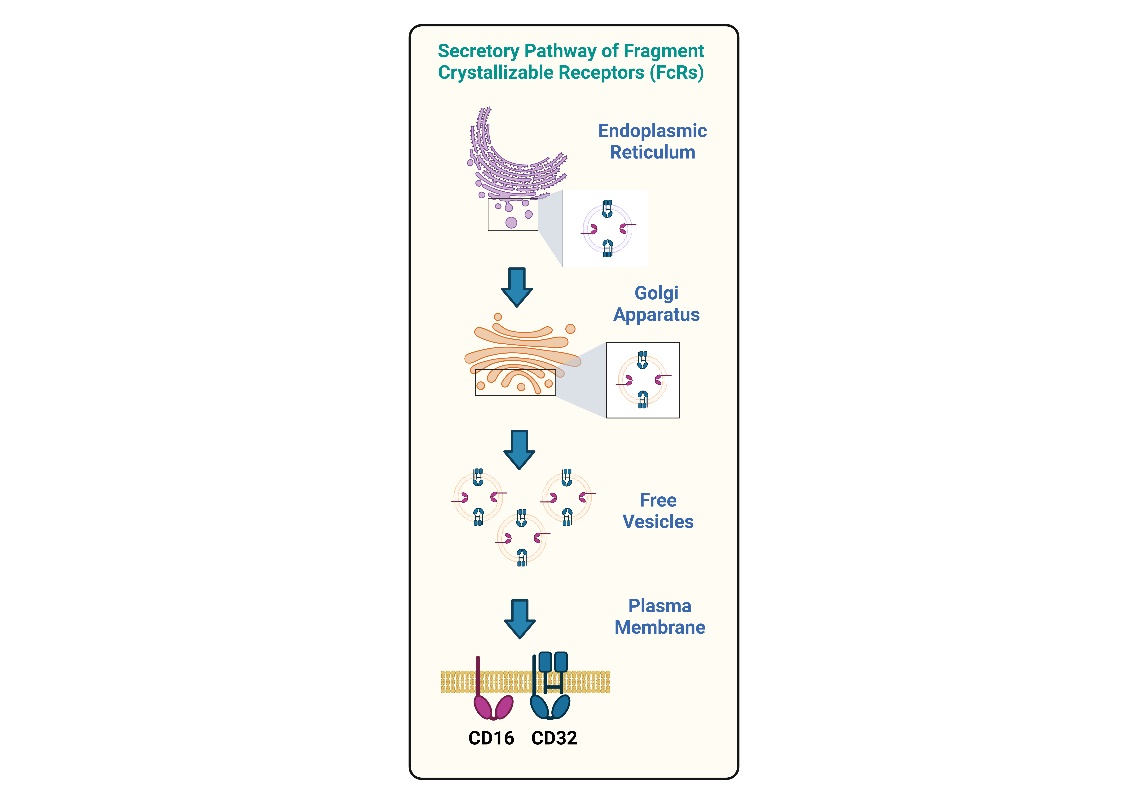

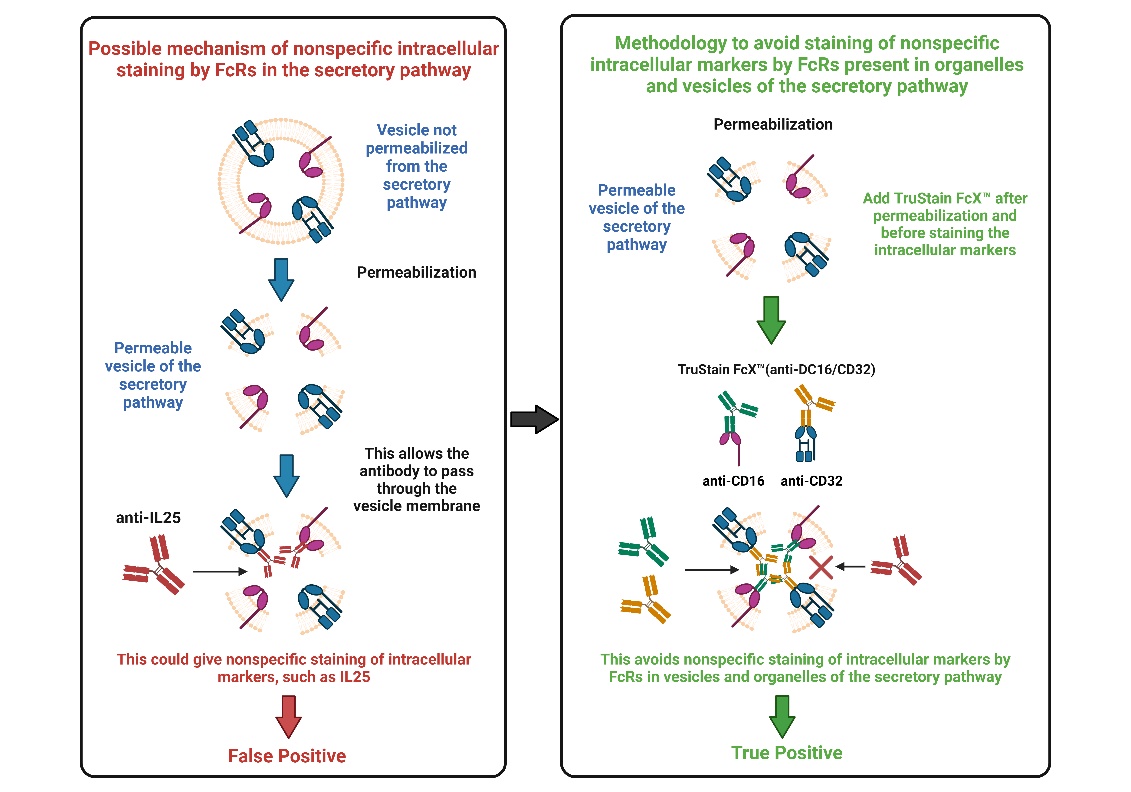
Supplementary Figure S7. IL-25 expression in lin^NEG^ TICs is true.** **A** Scheme of the secretory pathway followed by Fragment Crystallizable Receptors (FcRs). **B** Scheme of the possible non-specific staining mechanism for intracellular markers, such as IL25. This could be mediated by the presence of FcRs in organelles and vesicles of the secretory pathway. **C** Scheme of the methodology we used to avoid nonspecific staining associated with the presence of FcRs in organelles and vesicles of the secretory pathway. **D.** Cytometry analysis showing that lin^NEG^ TICs really express high amounts of IL25 despite intracellular blockade of FcRs with Trustain FcX^TM^ (n=2). FMO= Fluorescence minus one, FcRs= Fragment Crystallizable Receptors, TECs= Thymic Epithelial Cells, lin^NEG^ = Lineage negative cells.

**D**

**C**

**B**

**A**

**Supplementary tables**

**Table S1. Table with the raw value data of the number of TICs from Fraction 2 of the Thymus isolated in the 5 wash cycles.** After the cycle 5 ≤2, 500 TICs from the fraction 2 of the thymus were present in the supernatant.

|  | **Replicate Values** | | | | | |
| --- | --- | --- | --- | --- | --- | --- |
|  | **# of Thymic Interstitial Cells (TICs) from Fraction 2 of the Thymus** | | | | | |
| **Wash Cycle** | **Lobe 1** | **Lobe 2** | **Lobe 3** | **Lobe 4** | **Average per Thymic Lobe** | **Average per Thymus** |
| **1** | 645 000 | 200 000 | 772 500 | 368 750 | 496 562.5 | 993 125 |
| **2** | 60 000 | 22 500 | 150 000 | 175 000 | 101 875 | 203 750 |
| **3** | 70 000 | 11 250 | 40 000 | 5000 | 31 562.5 | 63 125 |
| **4** | 56 250 | 6250 | 15 000 | 0 | 19 375 | 38 750 |
| **5** | 5000 | 0 | 5000 | 0 | 2500 | 5000 |

**Table S2.** **Primary antibodies for Immunofluorescence staining and their dilutions**

| **Marker** | **Dilution** |
| --- | --- |
| anti-mouse CD80 PE/Cyanine7 (Cat. 104733) | 1:500 |
| Aire eFluor™ 660 (50593480) | 0.65:500 |
| CD326 Brilliant Violet™ 510 (1191155) | 0.65:500 |
| CD45 Alexa Fluor®700 (1386045) | 0.4:500 |
| CD11b PE (553311) | 0.4:500 |
| Podoplanin PE/Dazzle™594 (1237095) | 0.65:500 |
| Sca-1 PE-Cyanine7 (25598182) | 0.15:500 |
| CD146 PerCP-Vio700 (130103795) | 1.7:500 |
| Lgr5 PE-Vio770 (1301D1391) | 0.6:500 |
| CCL21 Alexa Fluor® 350 (IC457U-100UG) | 0.75:500 |
| IL-17E/IL-25 Alexa Fluor® (IC13991S-100UG) | 0.65:500 |

**Table 3. Comparison of the different methods described for obtaining clonogenic TECs, TECs and TICs.** NR= Not reported

|  | Ocampo *et al.* | Villegas *et al.* | Campinoti *et al.* | Campinoti *et al.* | Sekai *et al.* |
| --- | --- | --- | --- | --- | --- |
| **Year** | 2023 | 2018 | 2020 | 2020 | 2019 |
| **Type of cells obtained** | Clonogenic TECs, mature TECs and TICs | Clonogenic mature TECs, TECs and TICs | Clonogenic TECs and mature TECs | TICs | Clonogenic TECs and mature TECs |
| **Model** | Mouse | Human | Human | Human | Mouse |
| **Explant size** | ≤ 1 mm | ~ 10 mm |  | NR |  |
| **Enzymatic Treatment** | No | No | 0.4 mg/mL Collagenase D  0.6 mg/mL Dispase II  40μg/mL DNAse I  for ~30–45 min at 37 °C. | No | 0.5 U/mL Liberase  0.2 mg/mL DNase I  in RPMI media at 37 °C for 10 min with gentle agitation every 5 min . |
| **Petri Dish Diameter** | 70 mm | 75 mm |  | 60 mm |  |
| **Basal Medium** | DMEM/F12 | RPMI-16490 | cFAD medium (mixture 3:1 of DMEM and Ham's F-12) | Megacell medium | DMEM |
| **Supplement** | 20% FBS  1% penicillin‒streptomycin | 20% Horse Serum + 0.2% Ultroser G  2 mmol/l l-glutamine  100 IU/ml penicillin  100 μg/ml streptomycin | 10% Fetal Bovine Serum  1% penicillin‒streptomycin  0.4 μg/ml Hydrocortisone 10−10M Cholera Toxin  2 × 10−9 M Triodothyronine (T3)  5 μg/ml Insulin  10 ng/ml hEGF, at day 3 | 2.5% FBS HI  1% Penicillin/Streptomycin  1% L-glutamine  1% Non-Essential Aminoacids  100 mM beta-Mercaptoethanol  basic-FGF | 10% KnockOut Serum Replacement (KSR)  1% penicillin and streptomycin  0.4 μg/ml Hydrocortisone 10−10M Cholera Toxin  2 × 10−9M Triodothyronine (T3)  5 μg/ml Insulin  10 ng/mL EGF  10^3^ U/mL LIF |
| **Treatment of Plate** | No | No | No | Pre-coated with Matrigel^TM^ diluted 1:100 in Megacell medium | 0.1% gelatin |
| **Feeder Layer Cells** | No | No | Lethally irradiated mouse fibroblasts 3T3-J2 | No | STO cells were treated with10μgmL−1mitomycin-C |
| **Wash cycles to remove fraction 1 cells (thymocytes)** | Yes | Yes |  | Yes |  |
| **Wash to remove fraction 2 cells** | Yes | No |  | No |  |
| **Cells outgrown from the explants** | 5 days | 7-8 days | NA | 7 days |  |
| **Medium exchange** | 5 days | 3 days | NR | NR | 3 days |
| **TICs subtypes reported** | Thymic fibroblasts PDPN^+^  Hem lin^POS^ cells  Lin^NEG^ Aire^+^IL25^+^ cells | Thymic fibroblasts |  | Pericytes that express E7, VIM, PDGFRα, PDGFRβ, CD90, CD146, NG2 and ALP. |  |
